# Application of contrast-enhanced ultrasonography for gallbladder squamous cell carcinoma: a case report

**DOI:** 10.3389/fonc.2024.1502226

**Published:** 2024-12-11

**Authors:** Liqin Ruan, Xiaoyong Wu, Guiping Peng, Jing Zhang, Weili Chen

**Affiliations:** ^1^ Department of Hepatobiliary Surgery, Jiujiang City Key Laboratory of Cell Therapy, Jiujiang No.1 People’s Hospital Jiujiang, Jiujiang, Jiangxi, China; ^2^ Department of Ultrasound, Jiujiang City Key Laboratory of Cell Therapy, Jiujiang No.1 People’s Hospital Jiujiang, Jiujiang, Jiangxi, China; ^3^ Laboratory of Pathology, Jiujiang City Key Laboratory of Cell Therapy, Jiujiang No.1 People’s Hospital Jiujiang, Jiujiang, Jiangxi, China

**Keywords:** gallbladder, squamous cell carcinoma, case report, time-intensity curve, contrast-enhanced ultrasound

## Abstract

Preoperative diagnosis of Gallbladder squamous cell carcinoma (GBSCC) is difficult, and the contrast-enhanced ultrasound (CEUS) pattern has never been reported before. We present a case of GBSCC where CEUS revealed special findings that facilitated early diagnosis. CEUS demonstrated irregular peripheral ring-like enhancement during the arterial phase, with hypoenhancement in the late phases, and an irregular non-enhancing area persistently present in the center of the lesion.

## Introduction

GBSCC is a rare and aggressive malignancy with a poor prognosis. It is often identified at an advanced stage due to its nonspecific symptoms. The tumor tends to grow to a significant size before symptoms become apparent and is frequently detected incidentally during examinations for other conditions (1). Given the rarity of GBSCC, its CEUS imaging characteristics are not well established. In this report, we describe the CEUS imaging features of GBSCC. This is, to our knowledge, the first report detailing the CEUS findings in GBSCC cases.

## Case presentation

**Figure 1:**
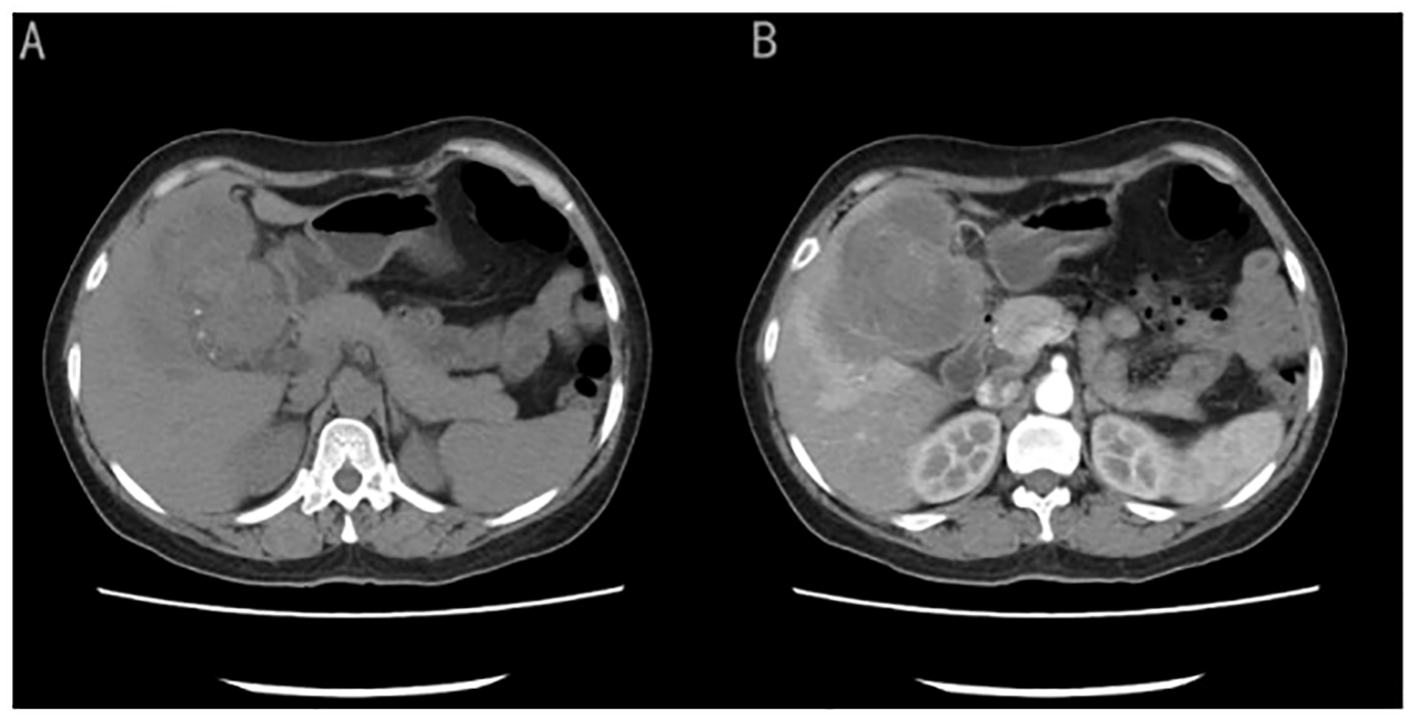
**(A)** CT image showed an irregularly shaped gallbladder with a mass measuring approximately 7.8cm × 7.4cm of mixed hypo-density). **(B)** On a contrast-enhanced CT scan showed slight heterogeneous enhancement of the mass.

**Figure 2:**
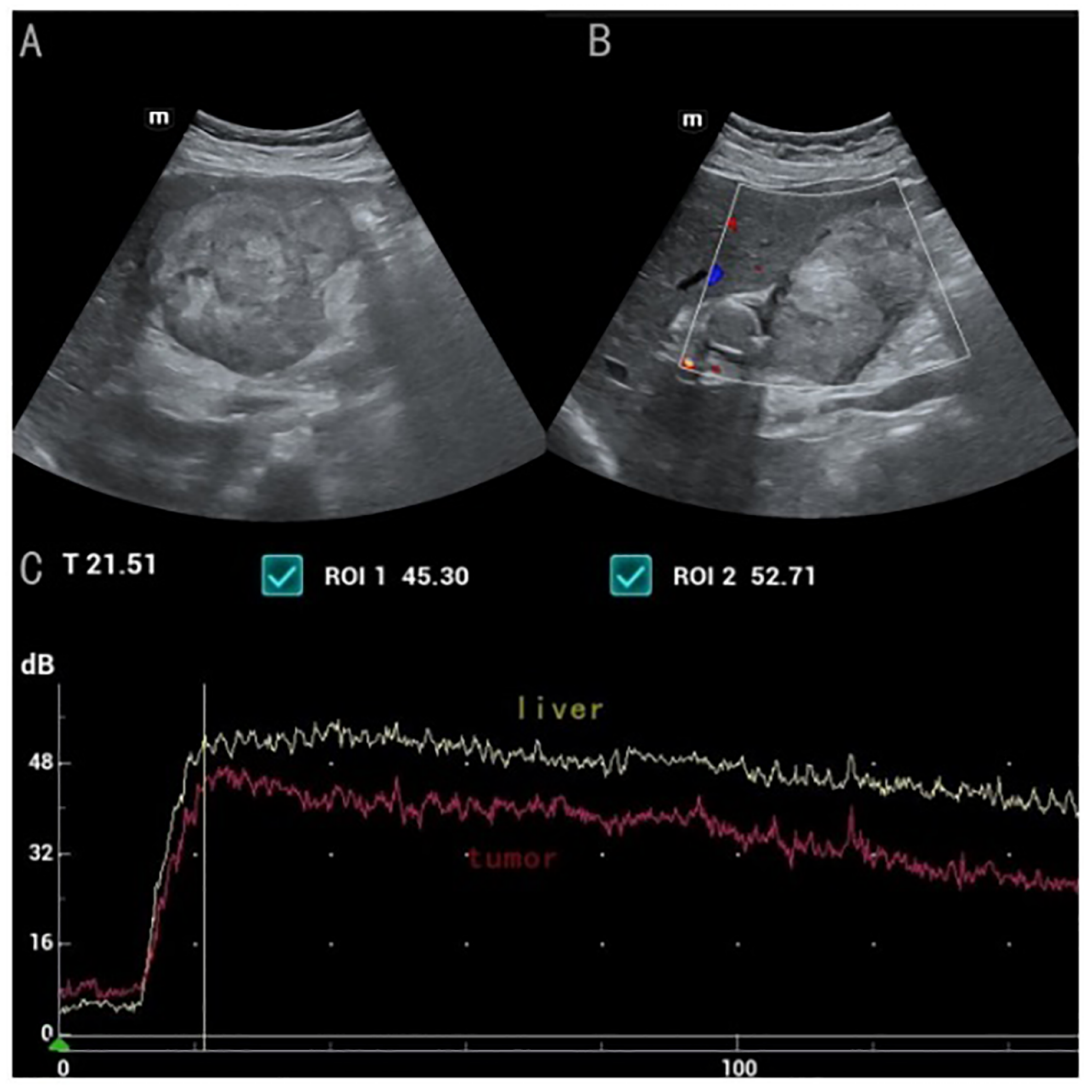
**(A)** the short-axis section of the hypoechoic mass showed a broad "onion" change **(B)** CDFI showed poor blood flow signals in the mass **(C)** Time-intensity curves displayed the wash-in time of 10s, TTP of 22s, The TIC from contrast-enhanced ultrasound shows that the AUC area of the mass is significantly smaller than that of the normal liver tissue.

**Figure 3:**
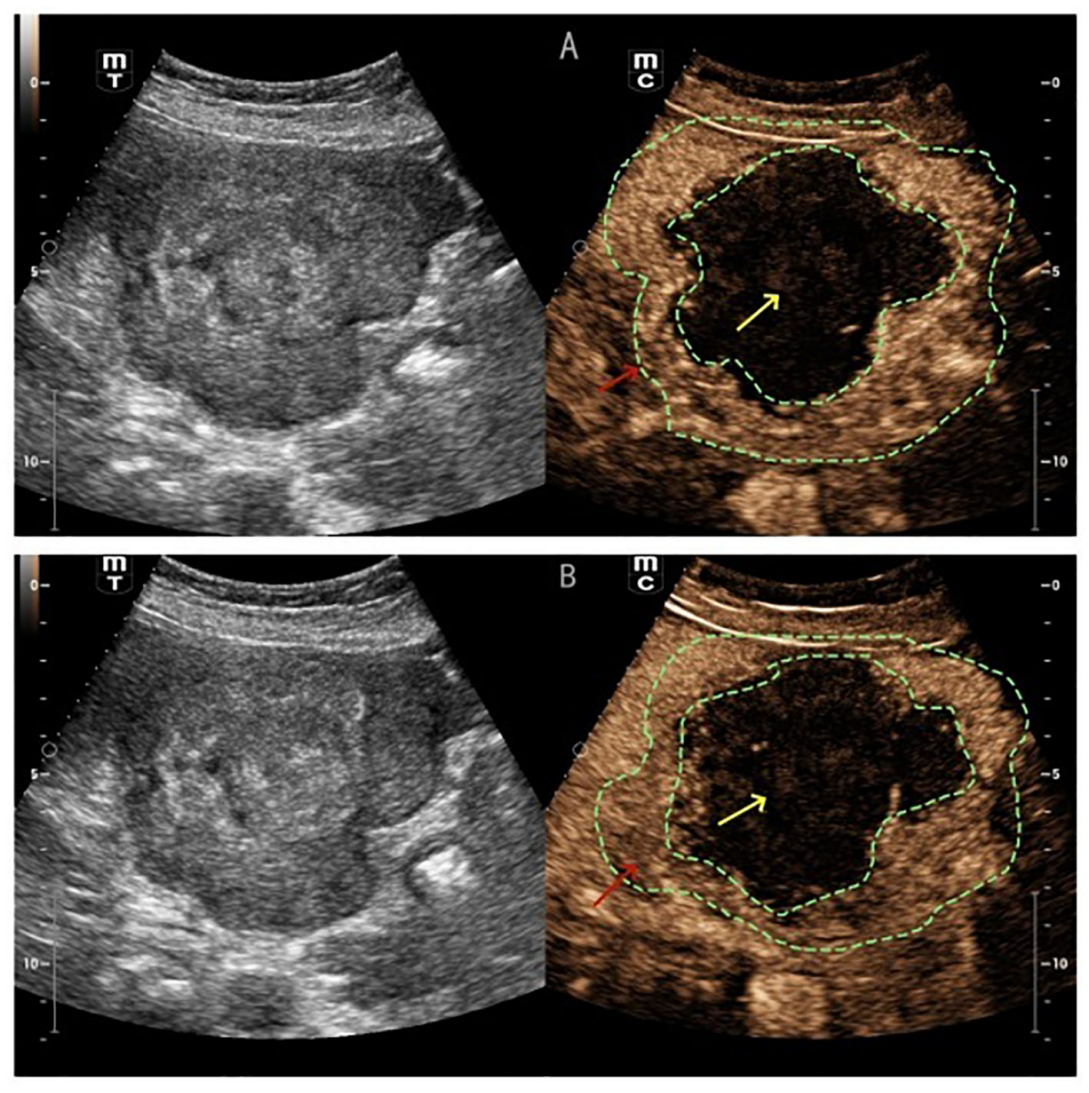
**(A)** CEUS showed the lesion periphery hyper-enhancement (red arrow) **(B)** the mass becomes hypo-enhancement in the late phase (red arrow), with an irregular non-enhancing area in the central region (yellow arrow).

A 50-year-old female patient presented with a chief complaint of persistent upper abdominal bloating and discomfort for a month. Laboratory test results, including assessment of tumor markers and routine blood tests, were within the standard levels. Abdominal computed tomography (CT) revealed that an irregularly shaped gallbladder had a mass measuring approximately 7.8cm × 7.4cm of mixed hypodensity (**Figure 1A**). On a contrast-enhanced CT scan, slight heterogeneous enhancement of the mass was noted.

(**Figure 1B**) Conventional ultrasound showed unclear boundaries and irregular contour of the gallbladder, measuring approximately 10.2cm×5.9cm, with a hypoechoic mass and multiple stones. the short-axis section of the hypoechoic mass showed an onion skin appearance (**Figure 2A**). The onion skin appearance is a particular pattern that appears as concentric layers of alternating hypoechogenicity and hyperechogenicity. The mass exhibited many malignant ultrasound features, such as solid components, hypoechogenicity, unclear boundaries and irregular shape. Color Doppler flow imaging (CDFI) showed poor blood flow signals in the mass (**Figure 2B**). Subsequently, CEUS was performed by injecting 2.4ml of ultrasound contrast agent (SonoVue, Bracco, Milan, Italy), followed by a flush with 5ml of 0.9% sterile saline through the antecubital vein. The examination was conducted at a low mechanical index of 0.083. The time-intensity curves (TICs) of the mass were calculated. CEUS demonstrated that blood flow into the tumor from the periphery in the early vascular phase at 12 s after the injection of agent, the mass began to be slowly enhanced from the periphery to the center at 10 s (wash-in time),the enhancement reached its peak [time to peak (TTP)] at 22 s, and the AUC of the mass is significantly smaller than that of the normal liver tissue (**Figure 2C**).The lesion periphery showed hyperenhancement, then, the mass becomes hypo-enhancement in the late phase, with an irregular non-enhancing area in the central region (**Figure 3**). Based on its conventional ultrasound and CT images, gallbladder malignancy was suspected, even though CEUS finding of the tumor in the late vascular phase and the CDFI presentation was atypical.

**Figure 4:**
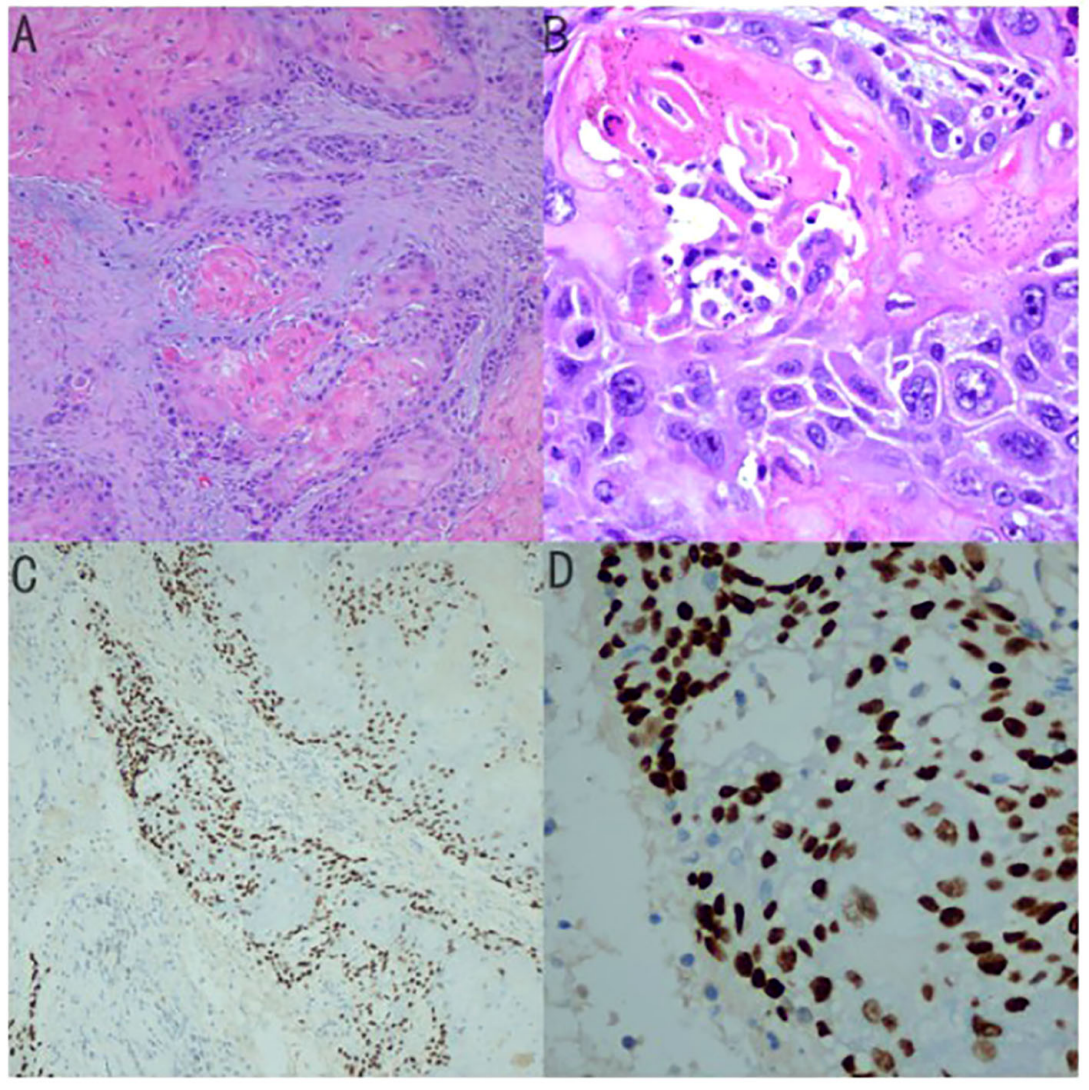
**(A)** Histological examination revealed that the gallbladder tumor had Keratin Pearls (magnification × 100) **(B)** magnification × 400 **(C)** Immunohistochemical tests for P40 (magnification × 100) **(D)** magnificationx 400.

The patient underwent palliative cholecystectomy for gallbladder cancer including resection of gallbladder in combination with partial liver resection. The tumor invaded the round ligament of the liver and the transverse colon, which could not be completely removed. Histological examination revealed that the gallbladder tumor had obvious palisade arrangement, intercellular bridges, or keratinization with a cancer pearl, serosal penetration, and liver tissue invasion (**Figure 4**). Immunohistochemical tests showed: MLH1(+), MSH2 (+), PMS2 (+), P40 (+), Her-2 (-), p53 (-), C-met (-). Final pathological finding was GBSCC. Postoperatively, the patient was treated with a combination of adjuvant cisplatin/gemcitabine (39 mg cisplatin and 1400 mg gemcitabine intravenous every 3 weeks) for four cycles. The patient is now in good condition. Written consent was obtained from the patient for publication of the case.

## Discussion

Although gallbladder cancer is the most common biliary tract malignancy, In 2021, it was estimated that the approximate incidence of all gallbladder and biliary tract cancers was 11,980 in United States (2). GBSCC is extremely uncommon, accounting for approximately 1 - 4 percent of all gallbladder malignancies (3).While the symptoms of GBSCC and gallbladder adenocarcinoma (GBAC) may be similar, GBSCC tends to be more larger, exhibit a higher degree of malignancy, and be at a more progressed stage. These more aggressive characteristics lead to a poorer overall survival rate for GBSCC compared to GBAC, even following a complete (R0) surgical resection (3, 4).

Conventional ultrasound proved to be very reliable and accurate in the diagnosis of gallbladder disease. CEUS of the gallbladder is an increasingly recognized modality that complements conventional ultrasound and cross-sectional imaging in the evaluation of neoplastic and non-neoplastic gallbladder conditions (5, 6). It is particularly valuable due to its ability to image microcirculation and provide optimal contrast resolution in real-time, which enhances diagnostic confidence (7). As a result of its rarity, the ultrasonography and CEUS imaging findings of GBSCC have seldom been published. Xanthogranulomatous cholecystitis (XGC) and GBAC are the main differential diagnoses for GBSCC.

Chen et al. reported that inhomogeneous enhancement in the arterial phase was the strongest independent predictor of malignancy, followed by interrupted inner layer, washout time ≤40 s, and wall thickness >1.6 cm (8). In the case of Xie et al., Carcinomas of the gallbladder typically exhibit hyperenhancement or isoenhancement during the early phase of contrast administration, fading out to hypo-enhancement within 35 s after contrast agent administration, and destruction of the gallbladder wall intactness was found in carcinoma (7). Serra, C. et al. found that the 60-second intralesional washout on CEUS is a helpful sign for malignancy but can yield false positives, particularly in smaller lesions (9). The indistinct boundary between the gallbladder wall and the liver was an independent predictor of malignancy. CDFI detected blood flow signals, and the irregular shape of the gallbladder, both of which were features of malignant lesions (10). Zhuang, B. et al. detected that an irregular shape, branched intralesional vessels and hypo-enhancement in the late phase were features indicating a malignant disease for gallbladder-confined focal tumors (11). XGC is a special type of cholecystitis (12), differentiating between XGC and gallbladder cancer with wall thickening can be particularly difficult (13–15). XGC manifests as thickening of the gallbladder wall, infiltrating into the liver and transverse colon (16, 17). CEUS has been recently reported to be a promising method in differentiating XGC from GBC, a continuous inner wall, hypoenhancement time greater than 80.5s, diffuse thickening, and hypoechoic nodules were valuable indicators in XGC (18).

In our case, GBSCC presented as a hypoechoic mass with irregular shape, and the unclear boundary between the gallbladder and peripheral liver parenchyma on ultrasound, which is consistent with gallbladder adenocarcinoma. Conventional ultrasound shows an onion skin appearance, which is commonly seen in testicular epidermoid cysts (19). The ultrasound finding exhibits a pathological correlation with alternating strata of compacted keratin and loosely dispersed desquamated squamous cells (20, 21). This observation aligns with our pathological analysis, which reveals the presence of squamous cell carcinoma and keratin. This may be indicative of a specific characteristic associated with this subtype of squamous cell carcinoma and represents a novel discovery. GBAC generally have abundant blood flow, which is different with our case. Squamous cell carcinoma (SCC) generally presents with larger tumors, and the rapid growth of the tumor leads to central ischemic necrosis. In CDFI, no significant blood flow is observed, and in CEUS examination, the central area of the tumor shows non-enhancing features, which is consistent with SCC. The CEUS findings are very similar to primary squamous cell carcinoma of the liver, with an irregular non-enhancing area also in the central region, which may be a characteristic manifestation of SCC (22). The low peak signal intensity, low AUC on CEUS and poor blood flow in CDFI were related to relatively few interstitial blood vessels in tumors. Compared to traditional ultrasound, CEUS provides information on tissue perfusion. In the case of the GBSCC, no significant blood flow was observed in conventional ultrasound, but CEUS results suggest abundant tissue perfusion.

## Conclusion

Our case presents the CEUS features of GBSCC, indicating that the TIC of GBSCC with a slow wash out time, low AUC and a lower peak signal intensity, which is different to GBAC. Additional evidence from further reports is needed to clarify the imaging characteristics and optimal treatment of GBSCC.

## Data Availability

The raw data supporting the conclusions of this article will be made available by the authors, without undue reservation.
